# Flt3/Flt3L Participates in the Process of Regulating Dendritic Cells and Regulatory T Cells in DSS-Induced Colitis

**DOI:** 10.1155/2014/483578

**Published:** 2014-10-13

**Authors:** Jing-Wei Mao, Yu-Shuang Huang, Hai-Ying Tang, Jian Bi, Yan-Fei Liu, Ying-De Wang

**Affiliations:** ^1^Department of Gastroenterology, the First Affiliated Hospital of Dalian Medical University, 222 Zhongshan Road, Dalian, Liaoning 116011, China; ^2^Department of Liver Diseases, Dalian Sixth People's Hospital, 269 Guibai Road, Dalian, Liaoning 116037, China

## Abstract

The immunoregulation between dendritic cells (DCs) and regulatory T cells (T-regs) plays an important role in the pathogenesis of ulcerative colitis (UC). Recent research showed that Fms-like tyrosine kinase 3 (Flt3) and Flt3 ligand (Flt3L) were involved in the process of DCs regulating T-regs. The DSS-induced colitis model is widely used because of its simplicity and many similarities with human UC. In this study, we observe the disease activity index (DAI) and histological scoring, detect the amounts of DCs and T-regs and expression of Flt3/Flt3L, and investigate Flt3/Flt3L participating in the process of DCs regulating T-regs in DSS-induced colitis. Our findings suggest that the reduction of Flt3 and Flt3L expression may possibly induce colonic immunoregulatory imbalance between CD103^+^MHCII^+^DCs and CD4^+^CD25^+^FoxP3^+^T-regs in DSS-induced colitis. Flt3/Flt3L participates in the process of regulating DCS and T-regs in the pathogenesis of UC, at least, in the acute stage of this disease.

## 1. Introduction

UC is a nonspecific inflammatory bowel disease (IBD) for which the etiology and pathogenesis are still not completely clear so far. One of the hypotheses regarding its pathogenesis involves the dysfunction of intestinal mucosal homeostasis due to a genetically determined miscommunication between commensal flora and intestinal immune system [1].

DCs are pivotal in tolerance induction and direct the differentiation of T cells [2]. Microbial antigen handled by DCs is believed to be of critical importance for immunity and tolerance in UC. Mucosal DCs play an important role in the development of oral tolerance, a phenomenon in which immune responses to a defined protein are blunted when the same protein has been orally fed before the rodents were systemically challenged [3]. The triggering of oral tolerance in patients with UC is impaired [4]. Interactions between DCs and T cells seem to be essential for the development of oral tolerance, in which CD4^+^CD25^+^FoxP3^+^T-regs that suppress systemic immune responses are primed [5]. T-regs have an essential role in maintaining immune tolerance in the gut and in the control of immune pathology in UC.

Recent studies demonstrated that there had been crosstalk between DCs and T-regs. Meanwhile, Flt3 and Flt3L were involved in this process [6, 7]. Flt3, a member of the tyrosine-kinase receptor family, was initially cloned from fetal liver cells with hematopoietic stem cell activity. Flt3L is the ligand for Flt3, which is a key regulatory cytokine for DCs commitment and development.

So far, very few studies have been performed regarding the participation of Flt3/Flt3L in the regulation of DCs and T-regs in UC. In this study, we aimed to clarify the role of Flt3/Flt3L in the immunoregulation between DCs and T-regs by detecting the colonic expression of Flt3/Flt3L and quantity of DCs and T-regs in terms of percentage in DSS-induced acute colitis.

## 2. Materials and Methods

### 2.1. Materials

Healthy male BALB/c mice weighing about 25 g were supplied by the Specific Pathogen Free (SPF) Laboratory Animal Center of Dalian Medical University. DSS was purchased from Sigma. FITC-conjugated anti-mouse CD103, PE-conjugated anti-mouse I-A/I-E (MHC-II), PE-conjugated anti-mouse CD4, PerCP-conjugated anti-mouse CD25, and PE-conjugated anti-mouse FOXP3 were supplied by BioLegend Company. Flt3 polyclonal antibody was supplied by Santa Company. The MaxVision TM plus Poly HRP (Mouse/Rabbit) IHC Kit was supplied by Beijing Zhongshan Goldenbridge Biotechnology Co., Ltd. Primers, PrimeScript RT Master Mix Perfect Real-Time kit, SYBR Premix Ex Taq (Tli RNaseH Plus), and DEPC were from Dalian, Takara Co., Ltd. ELISA kit for Flt3L was purchased from BIOVALUE Company.

### 2.2. Animals

A total of 20 BALB/c mice were randomly divided into two groups with 10 animals in each, the control group and DSS-induced colitis model group. Mice in the control group were fed with general diet and distilled water. The mice in model group were induced by oral administration of 50 g/L dextran sulphate sodium (DSS) solution every day for 7 days. Mice from two groups were sacrificed on day 7 and colonic tissue was collected for flow cytometry method (FCM), Real-time PCR, HE staining, and immunohistochemistry analysis. The blood samples of 0.8 mL from the heart were obtained for ELISA.

Mice were kept in a normally controlled breeding room with standard laboratory food and water for one week before the experiments. All experimental procedures were conducted according to the institutional guidelines for the care and use of Laboratory Animals of Dalian Medical University and conformed to the National Institutes of Health Guide for Care and Use of Laboratory Animals (Publication no. 85-23, revised in 1996). All protocols were approved by the Institutional Animal Care and Use Committee of Dalian Medical University.

### 2.3. Determination of DAI

Mice were weighed and checked for stool consistency and the presence of gross blood in the stool every day during the experiment. The DAI scores were assigned as follows: percentage of body weight reduction (0: no change; 1: 1–5%; 2: 6–10%; 3: 11–15%; 4: >15%); stool consistency (0: normal; 2: loose; 4: diarrhea); and the presence of fecal blood (0: normal; 2: positive occult blood test; 4: visible bleeding) [8]. The DAI was calculated as the sum of these scores divided by 3.

### 2.4. Determination of Histological Scoring

After mice were sacrificed, the entire colon was removed for gross colonic pathological damage observation. The colon samples with the worst damages were formalin-fixed, paraffin-embedded, and cut into 4 *μ*m thick sections for HE staining and histological analysis. Slides were examined and scored in a blinded fashion using the published grading system [9]. The scores were measured as follows: severity of inflammation (0: none; 1: slight; 2: moderate; and 3: severe), depth of injury (0: none; 1: mucosal; 2: mucosal and submucosal; and 3: transmural), and crypt damage (0: none; 1: basal one-third damaged; 2: basal two-thirds damaged; 3: only surface epithelium intact; and 4: entire crypt and epithelium lost).

### 2.5. Detecting DCs and T-Regs by FCM

The fresh clean colonic tissues about 0.2 g were mechanically disaggregated into a single cell suspension by using 75 *μ*m mesh. PBS-BSA-ETDA solution was added to the cells for a concentration of 1 × 10^6^/mL. The single cell suspension was centrifuged for 5 min at 1500 ×g at room temperature. After removing the supernatant, cells were incubated with 5 *μ*L PE-conjugated anti-mouse I-A/I-E and FITC-conjugated anti-mouse CD103 for 30 min at 4°C. The cells were then washed, centrifuged, and resuspended in 0.5 mL PBS before subjecting them to FCM for detecting DCs. For detecting T-regs, cells were stained with PE-conjugated anti-mouse CD4 and PerCP-conjugated anti-mouse CD25 for 30 minutes at 4°C. Cells were subsequently washed in staining buffer twice prior to fixation and permeabilization using eBioscience's buffers for intracellular staining of FoxP3 according to the manufacturer's instructions. The cells were then washed, centrifuged, and resuspended in 0.5 mL PBS before subjecting them to FCM.

### 2.6. Flt3 and Flt3L mRNA Expression by Real-Time PCR

Total RNA was extracted from colonic tissue samples using Trizol according to the manufacturer's protocols (Invitrogen). All RNA samples were checked for RNA quality by gel electrophoresis. Real-time PCR was performed by using SYBR Green I as a double-strand DNA-specific binding dye and continuous fluorescence monitoring. Reverse transcription (RT) reactions were performed using 0.5 *μ*L total RNA, 2 *μ*L PrimeScript RT Master Mix, and 7.5 *μ*L RNase Free dH_2_O. Amplification was performed in a total volume of 20 *μ*L containing 0.4 *μ*L of PCR forward primer and reverse primer, 0.4 *μ*L of ROX reference dye II, 10 *μ*L of SYBR Premix Ex Taq, 6.8 *μ*L of dH_2_O, and 2.0 *μ*L of DNA. An equal amount of cDNA from each sample was amplified using primers specific to each gene. The qPCR primer sequences for Flt3 were Flt3-F: GGTTTAAAGCGTACCCACGA and Flt3-R: CTCCAGGCCTCTCGTTCAC; for Flt3L were Flt3L-F: GAGGACGTCAACACCGAGAT and Flt3L-R: AGGTGGGAGATGTTGGTCTG; for GAPDH were GAPDH-F: CGTCCCGTAGACAAAATGGT and GAPDH-R: TTGATGGCAACCAATCTCCAC. The PCR reactions were done under the following conditions: 40 cycles of initial denaturation at 95°C for 30 s, denaturation at 95°C for 5 s, annealing at 60°C for 34 s, and extension at 72°C for 60 s. Melting curve analysis of amplification products was performed at the end of each PCR reaction. All qRT-PCR assays were performed in triplicate.

### 2.7. Measurement of Flt3 Protein Expression in Colonic Mucosa and Flt3L in Plasma by Immunohistochemistry and ELISA

Immunohistochemistry for Flt3 colonic mucosal expression was done according to the MaxVision TM plus Poly HRP IHC Kit protocol. Image analysis software (Image-Pro Plus 6.0) was used to measure the light density of positive control cells in which the cytoplasm was tan-yellow or brown after 3,3′-diaminobenzidine (DAB) staining. For each section, the positive integrated optical density (IOD) and total area of five representative visual fields without overlap were observed under high-power microscope (×400). The ratio of IOD and total area represented the mean value of optical density, with a higher ratio indicating a higher level of protein expression.

Flt3L protein expression in plasma was measured utilizing Flt3L ELISA kit following the manufacturer's instructions. Absorbance was observed and recorded under 450 nm in 30 minutes after termination reaction by ELISA. The corresponding concentration was found according to the absorbance values on the standard curve. A higher concentration indicates a greater quantity of protein expression.

### 2.8. Statistical Analysis

Data analysis was performed using SPSS version 18.0 (SPSS, Chicago, IL, USA) statistical software. Data showed a normal distribution and were expressed as means ± standard deviation. The results in different experimental groups were analyzed using one-way ANOVA. A *P* value of <0.05 indicates a statistically significant difference between different groups.

## 3. Results

### 3.1. Results of DAI and Histological Scoring

In the control group, mice behavior, physical status, diets, and defecation were normal. On the contrary, mice in the model group showed different degrees of lethargy, poor diets, purulent blood stool, and weight loss.

**Figure 1:**
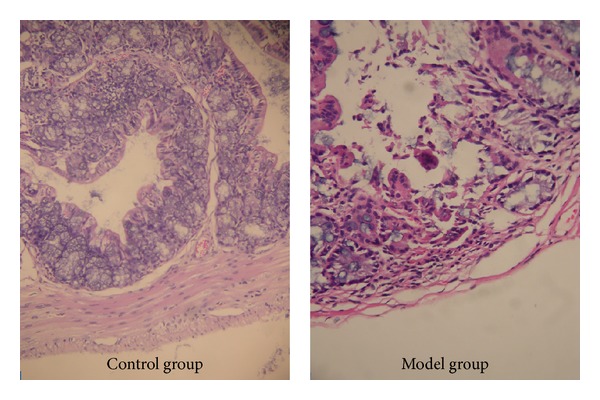
Pathological changes in the control group and UC model group on day 7 under light microscope (HE ×400). The colonic mucosa structure was intact in the control group. Mucosa and submucosa defects could be seen with infiltrations of inflammatory neutrophils and lymphocytes in the lamina propria in the UC model group.

Under light microscope, the colonic mucosa structure was intact in the control group. Mucosa and submucosa defects could be seen with infiltrations of inflammatory neutrophils and lymphocytes in the lamina propria in the model group (Figure 1).

The DAI and histological scoring in the model group were significantly higher than those in the control group (DAI: 3.15 ± 0.28 versus 0.00 ± 0.00, *P* < 0.05; histological scoring: 7.62 ± 0.40 versus 0.00 ± 0.00, *P* < 0.05).

### 3.2. The Amount of CD103^+^MHCII^+^DCs and CD4^+^CD25^+^FoxP3^+^T-Regs in Colonic Mucosa in terms of Percentage

**Figure 2:**
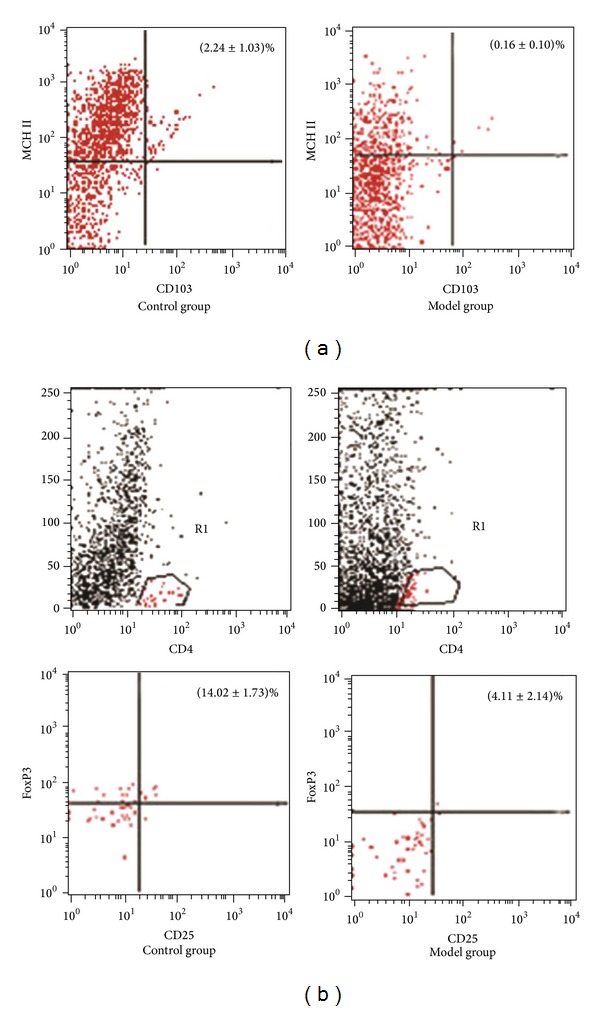
The percentage of CD103^+^MHCII^+^DCs and CD4^+^CD25^+^FoxP3^+^T-regs in the control and model group. (a) In the model group, the percentage of CD103^+^MHCII^+^DCs in the total colonic mucosal cells was significantly lower than that in the control group (0.16 ± 0.10 versus 2.24 ± 1.03, *P* < 0.05). (b) Compared with control group, the percentage of CD4^+^CD25^+^FoxP3^+^T-regs in CD4^+^T cells was significantly lower in the model group (4.11 ± 2.14 versus 14.02 ± 1.73, *P* < 0.05).

In the model group and the control group, the percentage of CD103^+^MHCII^+^DCs of total cells was (0.16 ± 0.10)% and (2.24 ± 1.03)%, respectively, and the percentage of CD4^+^CD25^+^FoxP3^+^T-regs in CD4^+^T cells was (4.11 ± 2.14)% and (14.02 ± 1.73)%, respectively. Statistical analysis showed that the percentage of both DCs and T-regs in model group decreased significantly compared to the control group (*P* < 0.05) (Figure 2).

### 3.3. Colonic Mucosal Flt3 and Flt3L mRNA Expressions in Different Groups

In the model group, expressions of Flt3 and Flt3L mRNA in the colonic mucosa were significantly lower than those in the control group (Flt3: 0.53 ± 0.06 versus 1.01 ± 0.13, *P* < 0.05; Flt3L: 0.18 ± 0.10 versus 1.01 ± 0.25, *P* < 0.05).

### 3.4. Protein Expressions of Flt3 in Colonic Mucosa and Flt3L in Plasma

**Figure 3:**
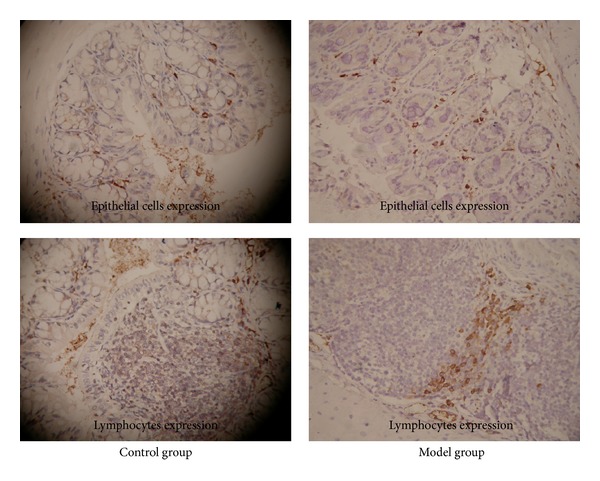
The colonic mucosal Flt3 expression in the control and UC model group. Immunohistochemistry showed that Flt3 was expressed in colonic epithelial cells and colonic lymphocytes. Expression of Flt3 protein in the colonic mucosa in the model group was significantly lower than that in the control group (31.66 ± 2.31 versus 82.19 ± 5.34, *P* < 0.05).

Immunohistochemistry showed that Flt3 was expressed in colonic epithelial cells and colonic lymphocytes. Expression of Flt3 protein in the colonic mucosa in the model group was significantly lower than that in the control group (31.66 ± 2.31 versus 82.19 ± 5.34, *P* < 0.05) (Figure 3).

The results of ELISA showed that the level of Flt3L in plasma in the model group was significantly lower than that in the control group (36.25 ± 6.35 versus 57.24 ± 5.97, *P* < 0.05).

## 4. Discussion

UC is a major clinical syndrome of IBD and is characterized by chronic mucosal inflammation of the colon. The etiology and pathogenesis of UC are not completely clear.

The DSS-induced colitis model is widely used because of its simplicity and many similarities with human UC. DSS can induce an acute colitis characterized by bloody stools, ulcerations, and infiltration of inflammatory cells [10]. In our study, the results showed that DAI and histological scoring in the model group were significantly higher than those in the control group. Histologically, DSS produces submucosal erosions, ulceration, and inflammatory cell infiltration as well as crypt abscesses.

It is apparent that the perturbation of the delicate imbalance of gut immune response plays a relevant etiological role in UC. Gut immune responses are normally regulated to maintain a state of mucosal tolerance, which represents a balance of the need to increase the protective immunity toward pathogens while not activating damaging inflammatory responses to harmless luminal antigens [11].

Recent studies suggested that DCs and T-regs are involved in the maintenance of immunological tolerance in the gastrointestinal tract [12, 13]. DCs, the most important antigen presenting cells (APC), are critically involved in the regulation of inflammatory response or immune tolerance, which mainly depends on different DCs subsets [14]. T-regs are a subpopulation of T cells including CD4^+^CD25^+^FoxP3^+^ T-regs, Tr1, Th3, and CD8^+^T-regs, all of which serve to suppress the immune system and maintain self-tolerance.

The amount of CD103^+^MHCII^+^DCs and CD4^+^CD25^+^FoxP3^+^T-regs in colonic mucosa in terms of percentage was measured by FCM in our study. The results showed that the percentage of CD103^+^MHCII^+^DCs and CD4^+^CD25^+^FoxP3^+^T-regs in model group decreased significantly compared to the control group. This result indicated that CD103^+^MHCII^+^DCs and CD4^+^CD25^+^FoxP3^+^T-regs were involved in the imbalance of colonic mucosal immunology. A previous study has demonstrated that interactions between DCs and T cells seem to be essential for the development of immune tolerance, in which CD4^+^CD25^+^FoxP3^+^T-regs are primed [5].

It was important for us to illustrate which pathway or factor was involved in regulating the balance between DCs and T-regs in the pathogenesis of UC. Latest study has found that there was a proposed homeostatic feedback loop between DCs and T-regs depending on Flt3/Flt3L; that is, increased numbers of DCs can result in an increased number of T-regs, and the increased T-regs can lead to a decrease in the number of DCs in a reverse way [15].

Flt3, a member of the tyrosine-kinase receptor family, was initially cloned from fetal liver cells with hematopoietic stem cell activity and was also expressed in DCs. Flt3L is the ligand for Flt3, which is a key regulatory cytokine for DCs commitment and development [16]. DCs, increased in number, can lead to expansion of T-regs by a mechanism of Flt3/Flt3L signal pathway that requires MHCII expression by DCs.

In this study, real-time PCR and immunohistochemistry were used to detect the Flt3 expression in colonic mucosa at the level of mRNA and protein. Our results indicated that expression of Flt3 in the inflamed colonic mucosa was significantly lower than that in the controls. Mucosal Flt3L mRNA and plasma Flt3L protein were measured by real-time PCR as well as ELISA. The results showed that Flt3L was significantly lower in the model group than that in the control group. It implied that the insufficient expression of Flt3 and Flt3L was related to the development of UC. This is in accordance with the previous conclusion of Collins et al. [17].

As a matter of course, Flt3/Flt3L signaling is well known for its role in hematopoiesis and particularly in DC ontogeny and is thought to contribute predominantly to DC differentiation. The increase in T-regs induced by DC expansion is sufficient to prevent some autoimmune diseases such as type 1 autoimmune diabetes and IBD.

However, the specific function of mucosal DCs and the definite regulatory mechanism between DCs and T-regs are still unknown and need to be clarified in future work.

In conclusion, the reduction of Flt3 and Flt3L expression may possibly induce colonic immunoregulatory imbalance between CD103^+^MHCII^+^DCs and CD4^+^CD25^+^FoxP3^+^T-regs in DSS-induced colitis. Flt3/Flt3L participates in the process of regulating DCs and T-regs in the pathogenesis of UC, at least, in the acute stage of this disease.
